# Development of a nomogram for membranous nephropathy prediction in patients with primary Sjögren’s syndrome: a 6-year retrospective study

**DOI:** 10.3389/fimmu.2024.1320880

**Published:** 2024-04-03

**Authors:** Lihui Guo, Shan Zhao, Xudong Liu

**Affiliations:** Department of Rheumatology and Immunology, The First Hospital of China Medical University, China Medical University, Shen Yang, China

**Keywords:** primary Sjögren’s syndrome, membranous nephropathy, nomogram, retrospective study, prognostic factor, rheumatoid factor

## Abstract

**Objectives:**

Nephritis is a life-threatening complication of primary Sjögren’s syndrome (pSS), with membranous nephropathy (MN) being prevalent. Renal biopsy is the gold standard for MN diagnosis, but it is invasive and cannot be repeatedly performed. This study aimed to develop a nomogram for the prediction of MN in patients with pSS.

**Methods:**

This retrospective study included patients with pSS admitted to the Rheumatology and Immunology Department of the First Affiliated Hospital of China Medical University between January 2015 and January 2021. A nomogram was developed using multivariable logistic regression analysis and evaluated using receiver operating characteristic (ROC) curve analysis. Bootstrap resampling analysis (1,000 times) was performed to evaluate the nomogram for discrimination and the calibration curve for consistency.

**Results:**

A total of 237 patients with pSS [aged 53.00 (44.00, 61.00) years] were included, with 35 pSS–MN patients. Based on clinical practice and multivariable logistic regression analysis, seven variables associated with pSS–MN were selected, including white blood cells, creatine, complement 3, rheumatoid factor, antinuclear antibodies, anti-SSA antibody, and interstitial lung disease. The area under the ROC curve was 0.860 (95% confidence interval: 0.796–0.919), indicating good predictive power. In addition, the nomogram exhibited excellent performance, as demonstrated by the calibration curve and decision curve analysis.

**Conclusion:**

This study developed a risk prediction nomogram for MN in patients with pSS, with high predictive power. It may be used to improve the management of patients with pSS.

## Introduction

Sjögren’s syndrome (SS) is a chronic autoimmune disorder characterized by exocrine gland dysfunction and dryness of mucosal surfaces (sicca symptoms), usually affecting the eyes and mouth (1–3). It may occur as a primary disorder (primary SS, pSS), but about one-third of patients have another underlying autoimmune condition (secondary SS), such as rheumatoid arthritis, systemic lupus erythematosus, scleroderma, or hypothyroidism (1–3). pSS is the second most common autoimmune disease, with a prevalence rate of 0.3%–1% (4, 5). pSS is more common in women (1, 3). It is commonly characterized by hypofunction of salivary and lacrimal glands, resulting in xerostomia and xerophthalmia (1–3). pSS can also include extra-glandular manifestations such as cutaneous vasculitis, nephritis, and interstitial lung disease (6). Membranous nephropathy (MN) is a glomerulonephritis characterized by subepithelial immune deposits containing antigen, immunoglobulin G (IgG), and complement components with little to no cellular proliferation or infiltration (7–10). It is a common cause of nephrotic syndrome in adults (7–10). MN is typically categorized as primary in the absence of another illness or exposure known to be etiologically related (7, 10). MN is typically categorized as secondary when a concurrent condition (e.g., infections, autoimmune disease, alloimmune disease, medications or toxins, or some malignancies) is present (7, 10, 11). Interstitial nephritis is the main manifestation of renal involvement in primary Sjögren’s syndrome rather than glomerular or vascular disease. With more and more multidisciplinary collaboration, we are finding more and more cases of primary Sjögren’s syndrome complicated with membranous nephropathy. In recent years, more and more case reports focus on the pSS-associated MN. Two recent studies showed that MN accounts for 36% and 50% of renal lesions in patients with pSS undergoing renal biopsy and is the main type of glomerulonephritis in pSS glomerulonephritis (12–14). MN remains one of the most common causes of nephrotic syndrome in nondiabetic adults, and its course is associated with reduced quality of life and increased mortality (11). Currently, renal biopsy and phospholipase A2 receptor (PLA2R) are used in the clinical diagnosis of MN, but these tools have significant drawbacks. Renal biopsy is invasive, and PLA2R is an uncommon test (15). The early screening of MN in high-risk patients with pSS using conventional clinical indicators is crucial for prompting diagnosis.

Therefore, this study aimed to establish a nomogram for the prediction of MN in patients with pSS.

## Methods

### Study design and patients

This retrospective study included patients with pSS admitted to the Rheumatology and Immunology Department of the First Affiliated Hospital of China Medical University between January 2015 and January 2021. The inclusion criteria were as follows: (1) ≥18 years, (2) pSS was diagnosed according to the 2002 American and European Consensus Group (AECG) criteria (16), and (3) underwent laboratory tests at least once during their initial hospital visit. The exclusion criteria were (1) patients with other connective tissue diseases, such as systemic lupus erythematosus, scleroderma, or vasculitis or (2) patients with hepatitis, malignancies, or other forms of nephritis. The study adhered to the principles of the Declaration of Helsinki. The study was approved by the Medical Science Research Institute of the First Affiliated Hospital of China Medical University Ethics Committee (no. 2019-216). The Ethics Committee waived the need for patients to provide written informed consent due to the nature of the retrospective study.

### Data collection and definition

The clinical data were collected from electronic medical records, including age, sex, white blood cell (WBC), lymphocyte (LY), neutrophilic granulocyte (NE), blood platelet (PLT), creatinine (CR), alkaline phosphatase (ALP), gamma-glutamyltransferase (GGT), albumin (ALB), immunoglobulin G (IgG), immunoglobulin A (IgA), immunoglobulin M (IgM), complement 3 (C3), complement 4 (C4), rheumatoid factor (RF), C-reaction protein (CRP), urinary albumin excretion rate (UAER), antinuclear antibody (ANA), anti-SSA antibody (SSA), Anti-Ro52 antibody (Ro52), proteinuria (PRO), urine occult blood (UOB), interstitial lung disease (ILD), interstitial lung disease (ILD) status, and EULAR Sjögren’s syndrome disease activity index (ESSDAI). All laboratory tests were performed using venous blood samples collected after overnight fasting. The investigators strictly adhered to clinical standard operating procedures (SOP) while inspecting items.

### Statistical analysis

R 4.1.1 (The R Project for Statistical Computing, www.r-project.org) and SPSS 24.0 (IBM, Armonk, NY, USA) were utilized for data analysis. The continuous data with a normal distribution were described as means ± standard deviations (SD) and analyzed using Student’s *t*-test; otherwise, they were presented as medians (interquartile range, IQR) and analyzed using the Wilcoxon rank-sum test. The categorical data were described as *n* (%) and analyzed using chi-square test or Fisher’s exact test. Univariable and multivariable logistic regression analyses were performed to identify independent risk factors for MN. A nomogram for predicting MN based on the results of the multivariable logistic regression analysis was developed and evaluated by 1,000 resampling bootstrap analyses.

Based on clinical practice, seven variables were selected from the variables with *P <*0.05 in the multivariable logistic regression analysis: WBC, CR, C3, RF, ANA, SSA, and ILD. Collinearity in the regression equation was assessed using the variance inflation factor, and the final model was obtained through stepwise regression and included WBC, CR, C3, RF, ANA, SSA, and ILD.

The accuracy of the nomogram model was evaluated using the area under the receiver operating characteristic curve (AUC-ROC). The concordance between the predicted and observed probabilities was determined through a calibration plot to assess the calibration of the nomogram model. Finally, a decision curve analysis (DCA) was conducted to test the model’s predictive performance. A two-sided *P*-value <0.05 was considered statistically significant.

## Results

**Figure 1:**
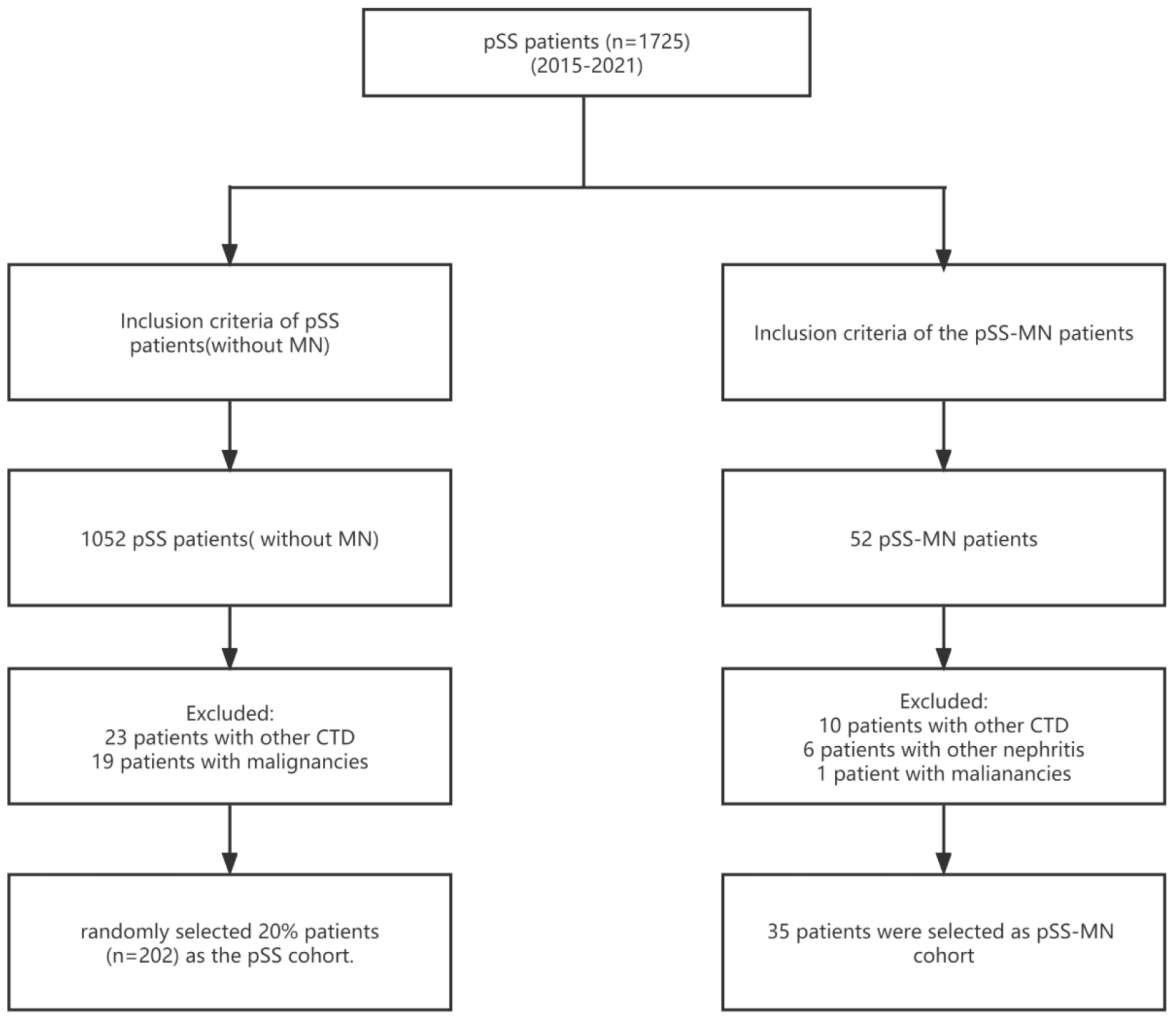
Patient flowchart.

A total of 1,725 patients with pSS were included; 33 patients with other CTD, 20 patients with malignancies, and six patients with other nephritis were excluded. Out of 1,010 patients who met the inclusion criteria for the pSS cohort, 35 patients with pSS–MN and 202 patients with pSS were finally included (**Figure 1**). All patients enrolled in pSS–MN underwent kidney biopsy. Light pathology revealed diffuse spike formation or uneven thickening of the basement membrane of glomerular capillaries. Immunofluorescence pathology revealed granular deposition of IgG and C3 in the walls of glomerular capillaries. Electron microscopy showed the deposition of electron density in glomerular visceral epithelial cells (podocytes) and diffuse fusion of podocytes’ foot processes, hence the diagnosis of membranous nephropathy. The baseline clinical data showed that the pSS–MN patients had significantly higher WBC, LY, NE, PLT, CR, C3, UAER, SSA, Ro52, PRO, UOB, and ILD and lower ALB, γ-globulin, IgG, C4, and RF than SS patients (all *P* < 0.05) (**Table 1**).

**Table 1:** Characteristics of the patients.

Variable	All	pSS (*n* = 202)	pSS–MN (*n* = 35)	p
*N*	237	202	35	
Age (years)	53.00 [44.00, 61.00]	53.00 [44.00, 61.00]	55.00 [40.00, 64.00]	
Sex				
Male	76 (32.1)	64 (31.7)	12 (34.3)	0.845
Female	161 (67.9)	138 (68.3)	23 (65.7)	
WBC (×10^9^/L)	4.63 [3.52, 6.05]	4.46 [3.31, 5.67]	5.81 [5.02, 6.52]	<0.001
LY (×10^9^/L)	1.40 [1.08, 1.83]	1.40 [1.02, 1.77]	1.57 [1.35, 2.04]	0.035
NE (×10^9^/L)	2.43 [1.78, 3.44]	2.24 [1.68, 3.24]	3.37 [2.95, 4.32]	<0.001
PLT (×10^9^/L)	209.00 [170.00, 246.00]	202.50 [167.00, 245.00]	226.00 [201.50, 276.50]	0.033
CR (µmol/L)	53.00 [47.00, 61.00]	52.00 [47.00, 59.00]	60.00 [50.50, 77.50]	0.002
ALP (U/L)	64.00 [51.00, 79.00]	63.00 [51.00, 79.00]	66.00 [54.50, 75.50]	0.451
GGT (U/L)	18.00 [13.00, 32.00]	18.00 [12.00, 31.75]	22.00 [15.50, 39.00]	0.214
ALB (g/L)	37.00 [32.50, 39.70]	37.60 [34.42, 40.27]	24.20 [19.45, 28.20]	<0.001
γ-Globulin (%)	23.40 [19.10, 29.10]	23.90 [20.20, 29.65]	18.30 [13.90, 25.00]	<0.001
IgG (g/L)	16.73 [12.19, 22.56]	17.28 [13.54, 23.34]	10.18 [6.64, 13.46]	<0.001
IgA (g/L)	2.91 [2.11, 3.82]	2.91 [2.04, 3.84]	2.73 [2.20, 3.35]	0.738
IgM (g/L)	1.19 [0.84, 1.89]	1.21 [0.86, 1.89]	1.07 [0.66, 1.56]	0.091
C3 (g/L)	0.92 [0.77, 1.06]	0.92 [0.76, 1.04]	1.08 [0.80, 1.23]	0.023
C4 (g/L)	0.17 [0.13, 0.21]	0.16 [0.13, 0.20]	0.23 [0.16, 0.29]	<0.001
RF (IU/mL)	23.80 [8.50, 78.80]	28.50 [8.62, 86.20]	12.90 [7.75, 23.15]	0.035
CRP (mg/L)	2.68 [1.90, 5.20]	2.70 [1.90, 5.52]	2.50 [1.92, 4.65]	0.437
UAER (g/24 h×10)	0.00 [0.00, 0.05]	0.00 [0.00, 0.00]	4.74 [2.46, 5.95]	<0.001
Anti-ANA				0.117
0 (negative)	105 (44.3)	82 (40.6)	23 (65.7)	
1 (positive +)	106 (44.7)	95 (47.0)	11 (31.4)	
2 (positive ++)	5 (2.1)	5 (2.5)	0 (0.0)	
3 (positive +++)	15 (6.3)	14 (6.9)	1 (2.9)	
4 (positive ++++)	6 (2.5)	6 (3.0)	0 (0.0)	
Anti-SSA				0.005
0 (negative)	76 (32.1)	72 (35.6)	4 (11.4)	
1 (positive +)	20 (8.4)	17 (8.4)	3 (8.6)	
2 (positive ++)	29 (12.2)	20 (9.9)	9 (25.7)	
3 (positive +++)	112 (47.3)	93 (46.0)	19 (54.3)	
Anti-Ro52				0.014
0 (negative)	64 (27.0)	58 (28.7)	6 (17.1)	
1 (positive +)	20 (8.4)	17 (8.4)	3 (8.6)	
2 (positive ++)	16 (6.8)	9 (4.5)	7 (20.0)	
3 (positive +++)	137 (57.8)	118 (58.4)	19 (54.3)	
PRO				<0.001
0 (negative)	162 (68.4)	162 (80.2)	0 (0.0)	
1 (positive +)	32 (13.5)	31 (15.3)	1 (2.9)	
2 (positive ++)	11 (4.6)	9 (4.5)	2 (5.7)	
3 (positive +++)	22 (9.3)	0 (0.0)	22 (62.9)	
4 (positive ++++)	10 (4.2)	0 (0.0)	10 (28.6)	
UOB				<0.001
0 (negative)	181 (76.4)	180 (89.1)	1 (2.9)	
1 (positive +)	26 (11.0)	13 (6.4)	13 (37.1)	
2 (positive ++)	24 (10.1)	9 (4.5)	15 (42.9)	
3 (positive +++)	6 (2.5)	0 (0.0)	6 (17.1)	
ILD				0.001
1 (none)	180 (75.9)	162 (80.2)	18 (51.4)	
2 (yes)	57 (24.1)	40 (19.8)	17 (48.6)	
ESSDAI	12.66 [0, 35]	12.88 [0, 35]	12.14 [5, 35]	0.377

Data are shown as average ± standard deviation, median (range), or n (%) as appropriate.

pSS, primary Sjögren’s syndrome; MN, membranous nephropathy; WBC, white blood cell; LY, lymphocyte; NE, neutrophilic granulocyte; PLT, blood platelet; CR, creatinine; ALP, alkaline phosphatase; GGT, γ-glutamyl transferase; ALB, albumin; IgG, immunoglobulin G; IgA, immunoglobulin A; IgM, immunoglobulin M; C3, complement 3; C4, complement 4; RF, rheumatoid factor; CRP, C-reactive protein; UAER, urinary albumin excretion rate; ANA, antinuclear antibody; SSA, anti-SSA antibody; Ro52, anti-Ro52 antibody; PRO, proteinuria: UOB, urine occult blood; ILD, interstitial lung disease; ESSDAI, EULAR Sjögren’s syndrome disease activity index.

**Figure 2:**
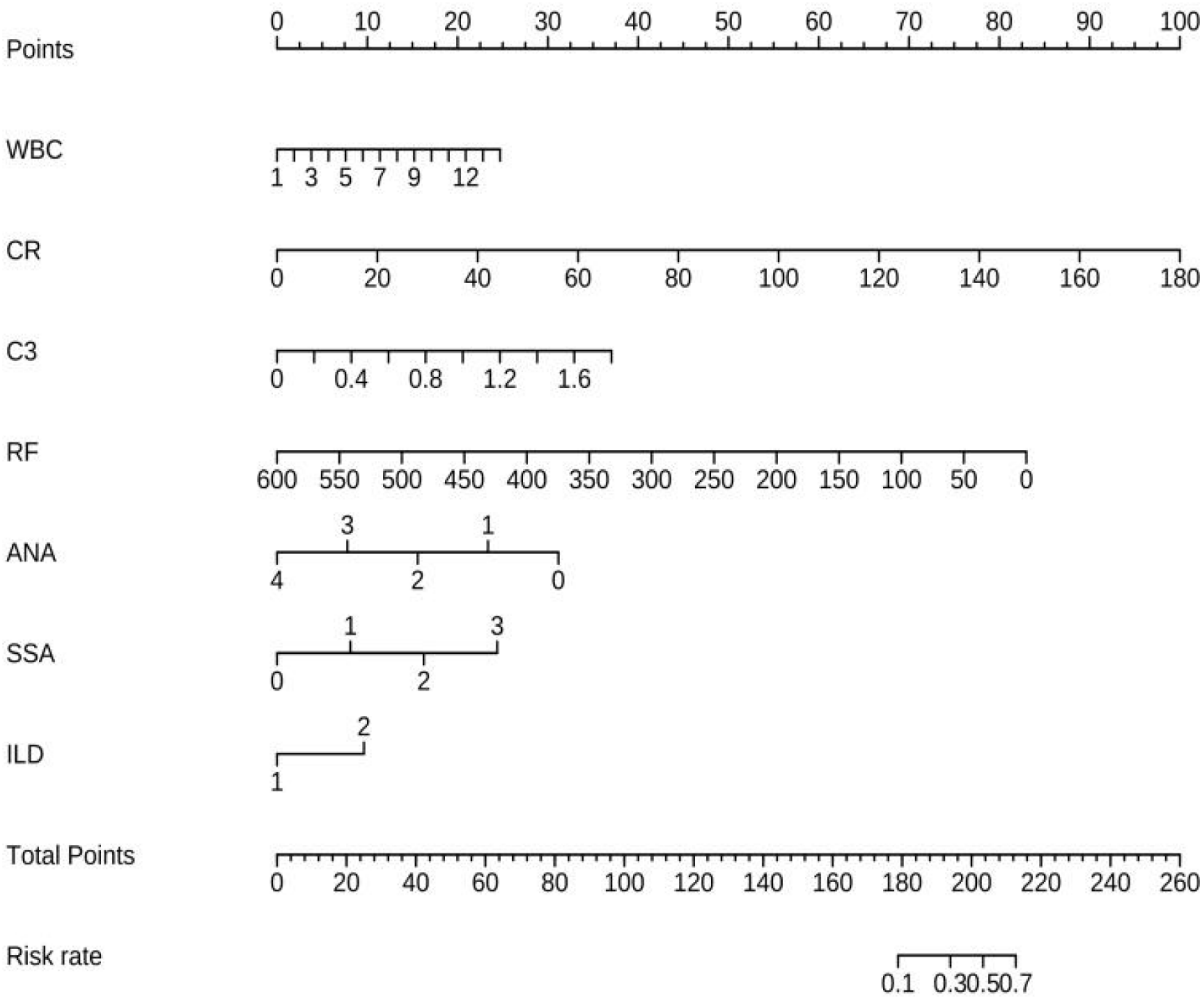
Model establishment and the nomogram.

Univariable logistic regression analysis revealed that WBC, NE, CR, ALB, γ-globulin, IgG, C3, RF, ANA, SSA, PRO, UOB, and ILD were associated with pSS–MN (all *P* < 0.05) (**Table 2**). Multivariable logistic regression analysis showed that CR (OR = 1.051, 95%CI: 1.025–1.078, *P* < 0.001), C3 (OR = 6.346, 95%CI: 1.026–39.243, *P* = 0.047), RF (OR = 0.988, 95%CI: 0.977–0.999, *P* = 0.029), and SSA (OR = 2.075, 95%CI: 1.373–3.136, *P* = 0.001) were independently associated with pSS–MN (**Table 3**). Based on clinical practice experience, WBC, CR, C3, RF, ANA, SSA, and ILD were selected to construct the nomogram model (**Figure 2**). The contribution of each variable to the outcome is displayed in **Supplementary Figure S1**. C3 exhibited the highest contribution among these variables.

**Table 2:** Univariable logistic regression analysis.

Names	*P*	OR	EXP(B)95%CI_LOW	EXP(B)95%CI_UP
WBC (×10^9^/L)	0.002	1.296	1.098	1.530
LY (×10^9^/L)	0.134	1.388	0.904	2.133
NE (×10^9^/L)	0.007	1.275	1.068	1.523
PLT (×10^9^/L)	0.555	1.001	0.998	1.003
CR (µmol/L)	0.001	1.039	1.015	1.063
ALP (U/L)	0.653	0.998	0.991	1.005
GGT (U/L)	0.677	0.999	0.994	1.004
ALB (g/L)	<0.001	0.762	0.705	0.824
γ-Globulin (%)	<0.001	0.885	0.830	0.943
IgG (g/L)	<0.001	0.737	0.661	0.823
IgA (g/L)	0.59	0.933	0.723	1.202
IgM (g/L)	0.292	0.811	0.548	1.198
C3 (g/L)	0.020	6.003	1.321	27.268
C4 (g/L)	0.985	1.004	0.659	1.530
RF (IU/mL)	0.037	0.992	0.984	1.000
CRP (mg/L)	0.169	0.955	0.895	1.020
UAER (g/24 h × 10)	<0.001	40.370	7.909	206.050
Anti-ANA	0.012	0.469	0.259	0.848
Anti-SSA	0.024	1.421	1.048	1.926
Anti-Ro52	0.436	1.119	0.843	1.485
PRO	<0.001	63.733	8.650	469.572
UOB	<0.001	11.872	6.176	22.821
ILD	<0.001	3.825	1.811	8.079
ESSDAI	0.394	0.893	0.734	1.102

WBC, white blood cell; LY, lymphocyte; NE, neutrophilic granulocyte; PLT, blood platelet; CR, creatinine; ALP, alkaline phosphatase; GGT, γ-glutamyl transferase; ALB, albumin; IgG, immunoglobulin G; IgA, immunoglobulin A; IgM, immunoglobulin M; C3, complement 3; C4, complement 4; RF, rheumatoid factor; CRP, C-reactive protein; UAER, urinary albumin excretion rate; ANA, antinuclear antibody; SSA, anti-SSA antibody; Ro52, anti-Ro52 antibody; PRO, proteinuria; UOB, urine occult blood; ILD, interstitial lung disease; ESSDAI, EULAR Sjögren’s syndrome disease activity index; OR, odds ratio; EXP(B)95%CI_LOW, lower bound of the 95% confidence interval; EXP(B)95%CI_UP, upper limit of the 95% confidence interval.

**Table 3:** Multivariable logistic regression analysis.

_	*P*	OR	EXP(B)95%CI_LOW	EXP(B)95%CI_UP
WBC (×10^9^/L)	0.100	1.186	0.968	1.453
CR (µmol/L)	<0.001	1.051	1.025	1.078
C3 (g/L)	0.047	6.346	1.026	39.243
RF (IU/mL)	0.029	0.988	0.977	0.999
Anti-ANA	0.052	0.497	0.246	1.005
Anti-SSA	0.001	2.075	1.373	3.136
ILD	0.062	2.373	0.958	5.878

WBC, white blood cell; CR, creatinine; C3, complement 3; RF, rheumatoid factor; ANA, antinuclear antibody; SSA, anti-SSA antibody; ILD, interstitial lung disease; OR, odds ratio; EXP(B)95%CI_LOW, lower bound of the 95% confidence interval; EXP(B)95%CI_UP, upper limit of the 95% confidence interval.

**Figure 3:**
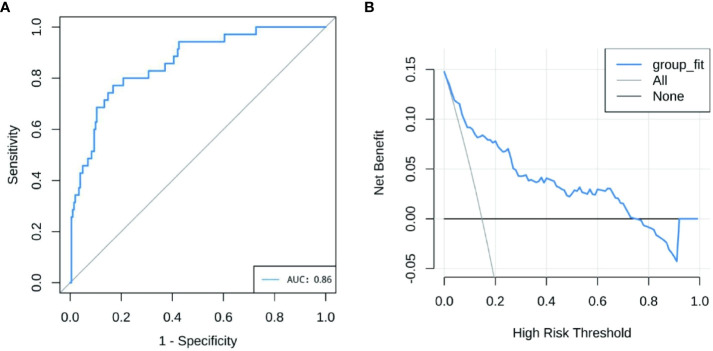
**(A)** Receiver operating characteristics curve of nomogram model. The X-axis is the sensitivity of the model to predict membranous nephropathy (MN), and the Y-axis is the specificity of the model to predict MN. **(B)** Decision curve analysis for the nomogram model. The Y-axis measures net benefit.

**Figure 4:**
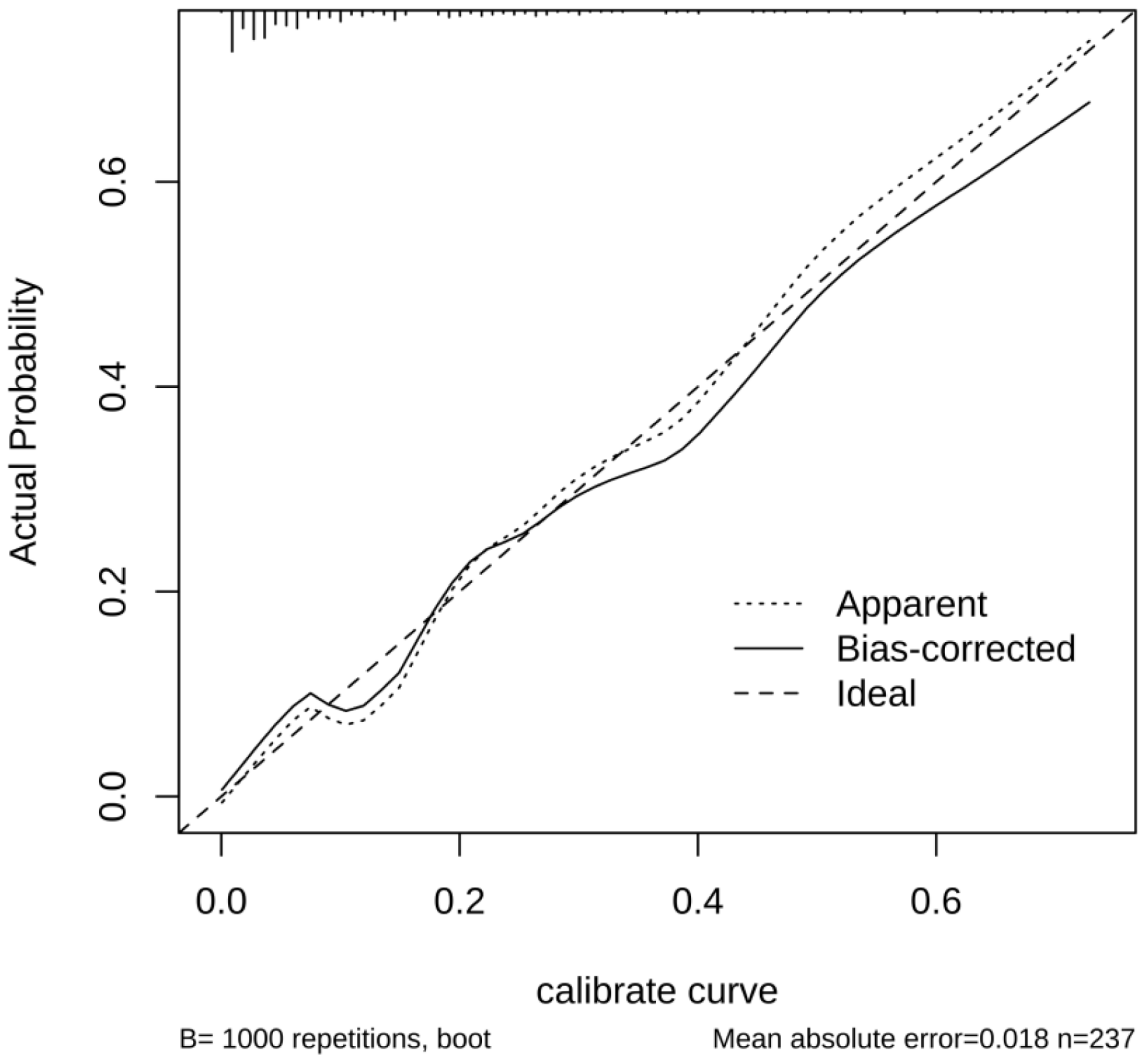
A calibration curve to evaluate the accuracy of the model.

The performance of the nomogram model by resampling it 1,000 times using the bootstrap method was evaluated, and the nomogram achieved an AUC of 0.860 (95% CI: 0.794–0.925) (**Figure 3A**). The DCA demonstrated the net benefit of using the nomogram model for predicting MN risk in patients with pSS (**Figure 3B**). When considering threshold probabilities between 0.1 and 0.7 for physicians or patients, the model provided greater net benefit compared with appropriate treatment strategies or no treatment at all. The calibration curve with 1,000 resampling showed a strong consistency between the predictions and actual observations of the pSS–MN risk prediction model (**Figure 4**).

## Discussion

This study developed and validated a risk prediction nomogram for MN in patients with pSS. The results may help improve the management of pSS by providing a simple-to-use tool to evaluate the risk of MN without the need for invasive procedures.

Previous studies tried to create predictive models for primary MN in various patient populations, achieving AUCs of 0.68–0.95 and based on various parameters: hypertension, chronic tubulointerstitial injury, 24-h proteinuria, and serum uric acid (AUC = 0.83) (17); age, estimated glomerular filtration rate (eGFR), and proteinuria (AUC = 0.83) (18); age, proteinuria, sPLA2R-Ab, and urinary α1-microglobulin corrected by creatinine (Uα1m/Cr) (AUC = 0.89) (19); persistent proteinuria and creatinine clearance (AUC not reported) (20); urinary β2-microglobulin (Uβ2m) and urinary excretions of IgG (AUC not reported) (21); neutrophil-to-lymphocyte ratio (AUC = 0.68) (22); aPLA2R-Ab, creatinine, urea, 24-h proteinuria, HDL-C, and neutrophils (AUC = 0.91) (23); sPLA2R-Ab at baseline and 3 months (AUC = 0.95) (24). Some of these models are still based on non-routine laboratory measurements (at least in some centers, especially in developing countries). In addition, these models were not specific to patients with pSS. Therefore, this study developed and validated a risk prediction nomogram for MN in patients with pSS using WBC, CR, C3, RF, ANA, SSA, and ILD. The nomogram had an AUC of 0.86, indicating a good predictive ability. The nomogram may help improve the management of pSS by providing a simple-to-use tool to evaluate the risk of MN without the need for invasive procedures.

The RF is an autoantibody against the Fc portion of IgG and is a hallmark of many autoimmune diseases, although it can also occur in healthy individuals. Approximately 60% of the patients with pSS are RF-positive (25, 26). RF has some predictive value for salivary glands, joint, and lung damage, chronic anemia, and secondary lymphoma in patients with pSS (27). Nevertheless, there is no report on the predictive value of RF for pSS–MN. The present study suggests that RF is a protective factor for MN, with its predictive effect opposing that of other types of organ damage in patients with pSS. Patients with pSS and elevated RF are younger and more likely to be positive for SSA and anti-SSB antibody (SSB), with high serum erythrocyte sedimentation rate (ESR), CRP, and IgG levels, indicating disease activity (28). Thus, younger and more active patients with pSS have a lower risk of developing MN. Although PLA2R is considered to be associated with MN in a previous study [van de Logts AE, 2019 #50], this study reviewed 35 cases of SS-MN and found that only 16 cases had PLA2R test, and only five cases were positive and 11 cases were negative. Therefore, PLA2R does not seem to be a specific indicator of SS-MN.

The complement system comprises more than 30 plasma proteins and cell surface receptors (29, 30). Previous studies reported the involvement of the complement system in systemic lupus erythematosus (SLE), with low levels of complement proteins (C3 and C4) serving as diagnostic markers for SLE and used to monitor disease activity (31, 32). A retrospective study comparing the clinical and serological features of secondary SS in patients diagnosed with SLE (SLE-SS) and SLE only found that patients with SLE-SS had a lower frequency of renal disease and hypocomplementemia of C3 and C4 (33). The present study found an association between low C3 and a lower risk of MN, which is consistent with previous research findings. In addition, of all the model variables, C3 contributed the most to the outcome.

A study analyzed the clinical features of a large cohort of Spanish patients with pSS. Patients with severe systemic disease (including lymphadenopathy, central nervous system, peripheral nervous system, pulmonary, renal, cutaneous, articular, hematological, and muscular damage) had a higher frequency of RF and low C3 and C4 levels (34). This Spanish study encompassed multiple systems and organs, while the present study focused primarily on pSS–MN and observed that a lower frequency of RF and higher C3 levels were associated with an increased risk of MN.

ANA, SSA, SSB, and RF are the four classic autoantibodies of pSS. ANA positivity occurs in 59%–85% of patients with pSS, whereas SSA and SSB occur in 50%–70% of patients with pSS and are associated with prolonged disease stage, extra glandular damage, and increased lymphocytic infiltration on glandular biopsy (35, 36). In the model developed here, SSA was a risk factor for pSS–MN, consistent with previous studies. ANA was a protective factor for pSS–MN, which has yet to be reported.

Furthermore, the model considers WBC as a risk factor for pSS–MN. WBCs are often associated with infection, and leukopenia may occur in patients with pSS who suffered from blood system involvement. Therefore, it could be hypothesized that SS patients with pSS with infection were more prone to develop MN, whereas those with hematologic involvement had a lower risk of MN.

Creatinine CR levels are used to evaluate renal function, and elevated creatinine CR indicates impaired renal function. The nomogram revealed that the risk of MN increased with within-normal-range creatinine CR levels.

Interstitial lung disease (ILD) is a common form of lung involvement in pSS patients with pSS, with a prevalence of 3%–60%, depending on the method used to identify ILD (37, 38). The results indicated an increased risk of MN in patients with pSS and ILD, suggesting the consistent involvement of kidneys and lungs in patients with pSS.

Although well designed, this study has some limitations. The retrospective analysis involved single-center data and a relatively small sample size of patients due to its low morbidity of pSS–MN, which may affect the model’s generalizability. In addition, only the variables available in the charts could be analyzed. At the same time, it could also be a strength since only variables routinely assessed in the clinical setting were included.

In conclusion, this study developed and validated a risk prediction nomogram for MN in patients with pSS. Future studies on large sample sizes and multicenter data should help fine-tune the predictors and optimize the model’s performance for pSS–MN prediction.

## Data availability statement

The original contributions presented in the study are included in the article/**Supplementary Material**. Further inquiries can be directed to the corresponding authors.

## Ethics statement

The studies involving humans were approved by the Medical Science Research Institute of the First Affiliated Hospital of China Medical University Ethics Committee (No. 2019-216). The studies were conducted in accordance with the local legislation and institutional requirements. The participants provided their written informed consent to participate in this study.

## Author contributions

LG: Writing – original draft. SZ: Writing – review & editing. XL: Writing – review & editing.

## Supplementary material

Supplementary Figure 1 Contribution of each variable to the outcome variable.
