# Supplementary figures and images for: Development of a nomogram for membranous nephropathy prediction in patients with primary Sjögren’s syndrome: a 6-year retrospective study
Source: Front Immunol. 2024 Apr 3;15:1320880. doi: 10.3389/fimmu.2024.1320880 (PMC11021693; doi:10.3389/fimmu.2024.1320880)

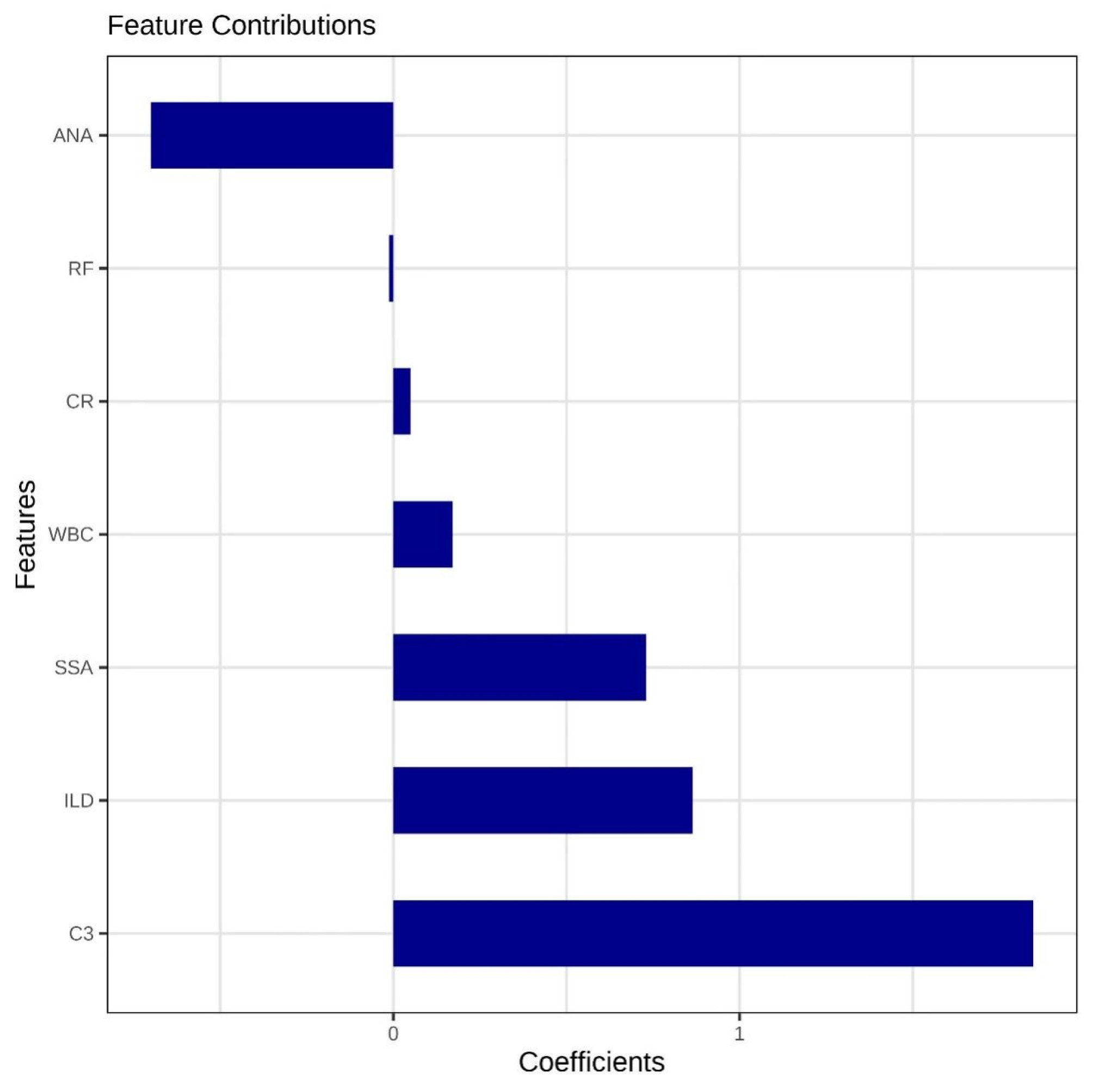

Supplement: Supplementary Figure 1 — Contribution of each variable to the outcome variable. [file Image_1.tif]
